# Supplementary material for: Antibacterial Activity of Electrospun Polyacrylonitrile Copper Nanoparticle Nanofibers on Antibiotic Resistant Pathogens and Methicillin Resistant Staphylococcus aureus (MRSA)
Source: Nanomaterials (Basel). 2022 Jun 22;12(13):2139. doi: 10.3390/nano12132139 (PMC9268565; doi:10.3390/nano12132139)

Supplementary Information

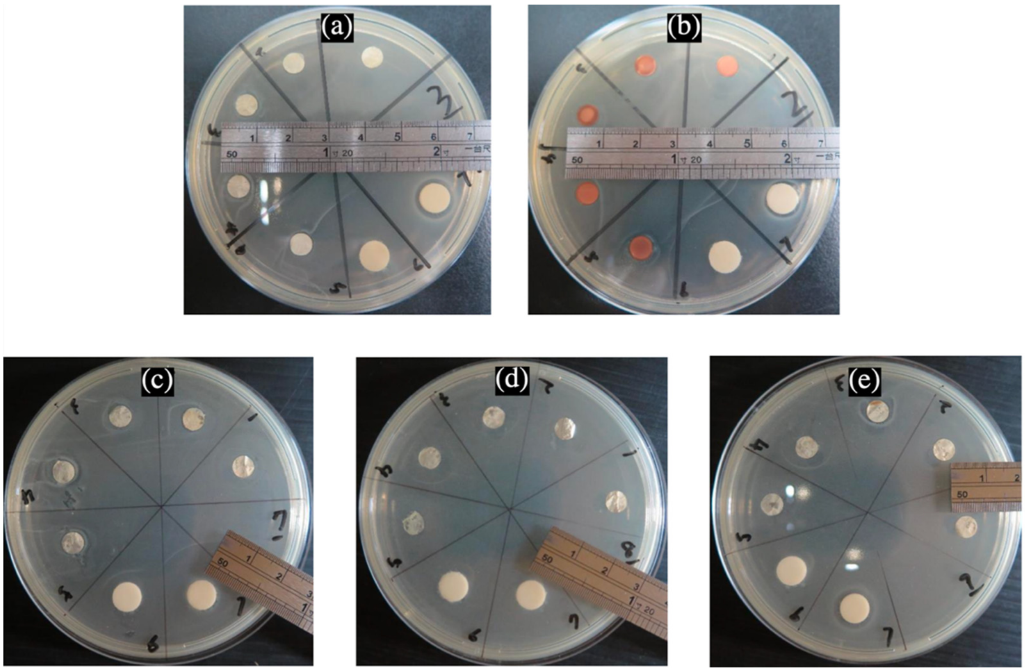

Figure S1. (**a**) Pure PAN Nanofiber Disk, (**b**) Bulk Cu Disk, (**c**) 5% PAN-CuNP Nanofiber Disk, (**d**) 10% PAN-CuNP Nanofiber Disk, and (**e**) 15% PAN-CuNP Nanofiber Disk ZOI Tests on *E. coli*, with 5 trials each labelled as sections 1-5. Section 6 is a negative control disk while Section 7 is a positive control disk (ampicillin).

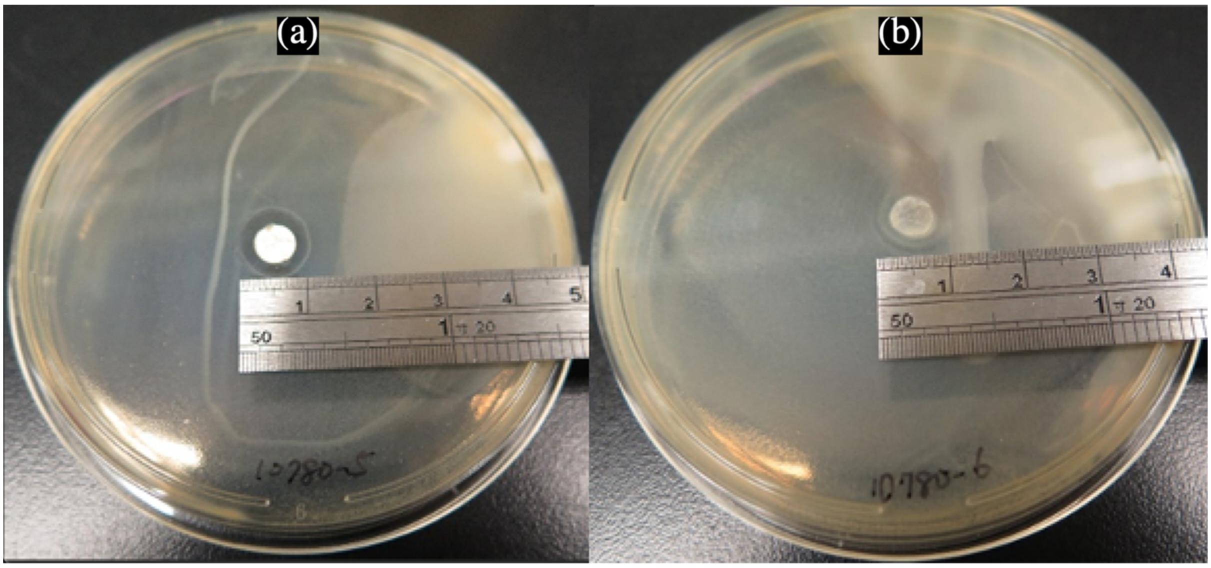

Figure S2. (**a**) 10% PAN-CuNP Nanofiber Disk and (**b**) 15% PAN-CuNP Nanofiber Disk ZOI Tests on *S. aureus* (*10780*)

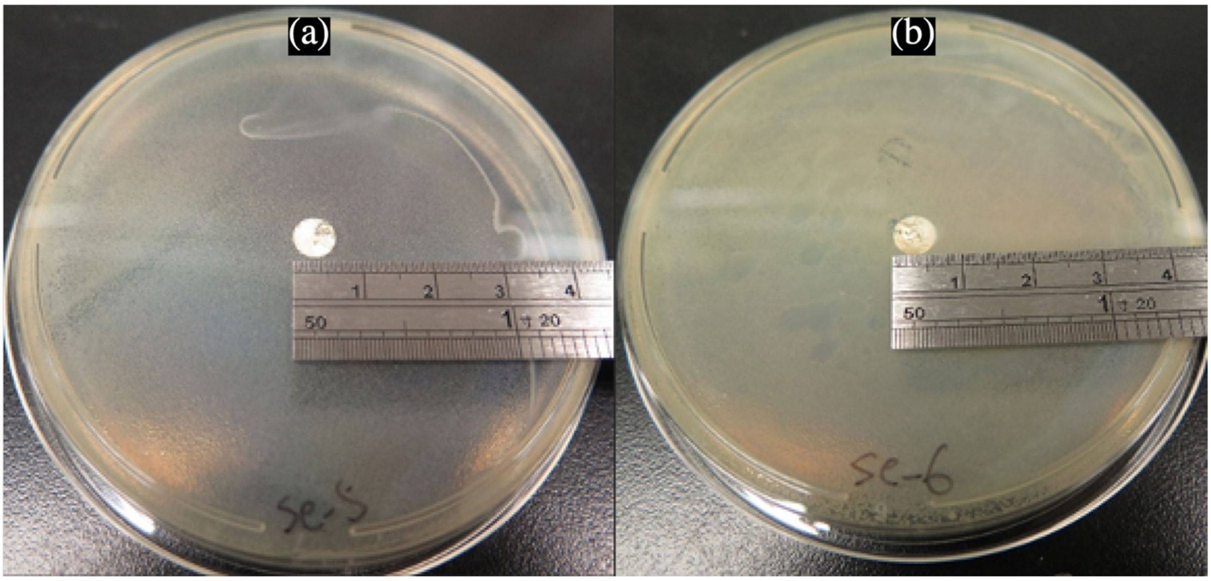

Figure S3. (**a**) 10% PAN-CuNP Nanofiber Disk and (**b**) 15% PAN-CuNP Nanofiber Disk ZOI Tests on *S. epidermidis*

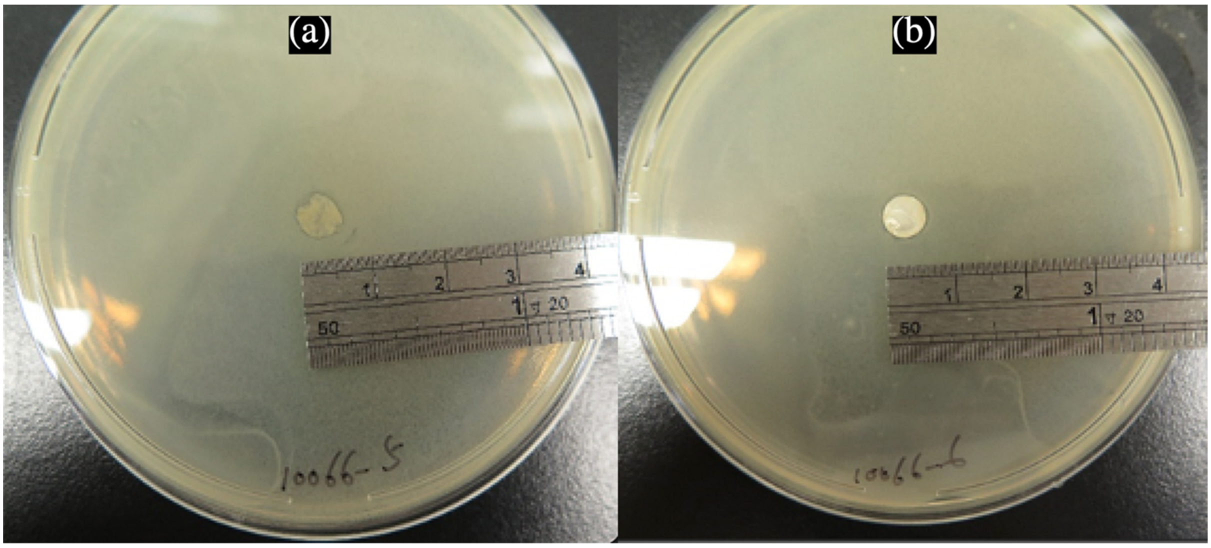

Figure S4. (**a**) 10% PAN-CuNP Nanofiber Disk and (**b**) 15% PAN-CuNP Nanofiber Disk ZOI Tests on *E. faecalis* (*10066*)

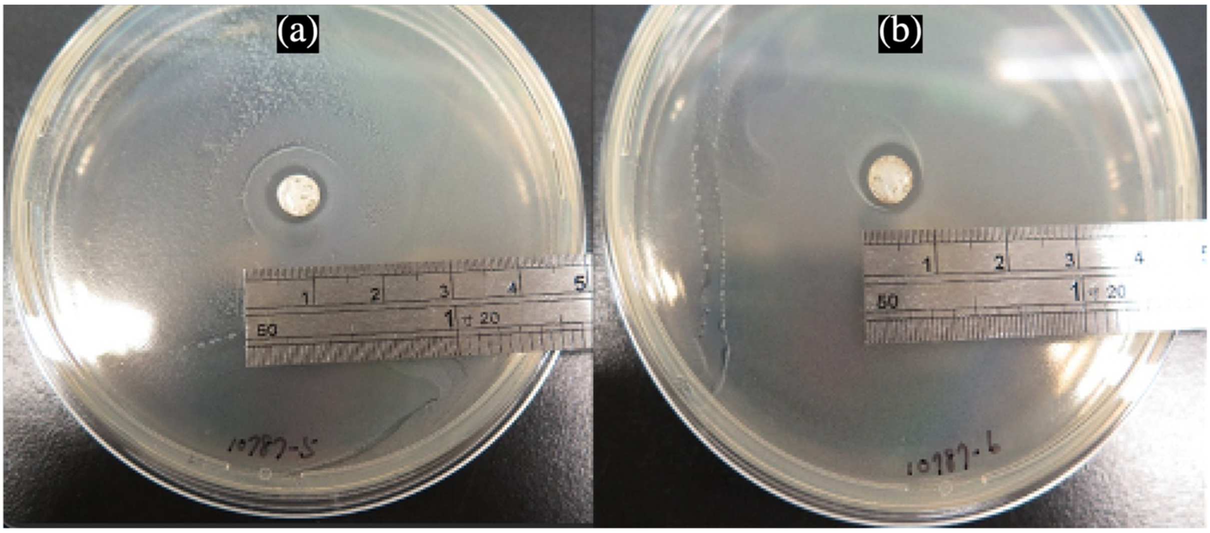

Figure S5. (**a**) 10% PAN-CuNP Nanofiber Disk and (**b**) 15% PAN-CuNP Nanofiber Disk ZOI Tests on *S. agalactiae* (*10787*)

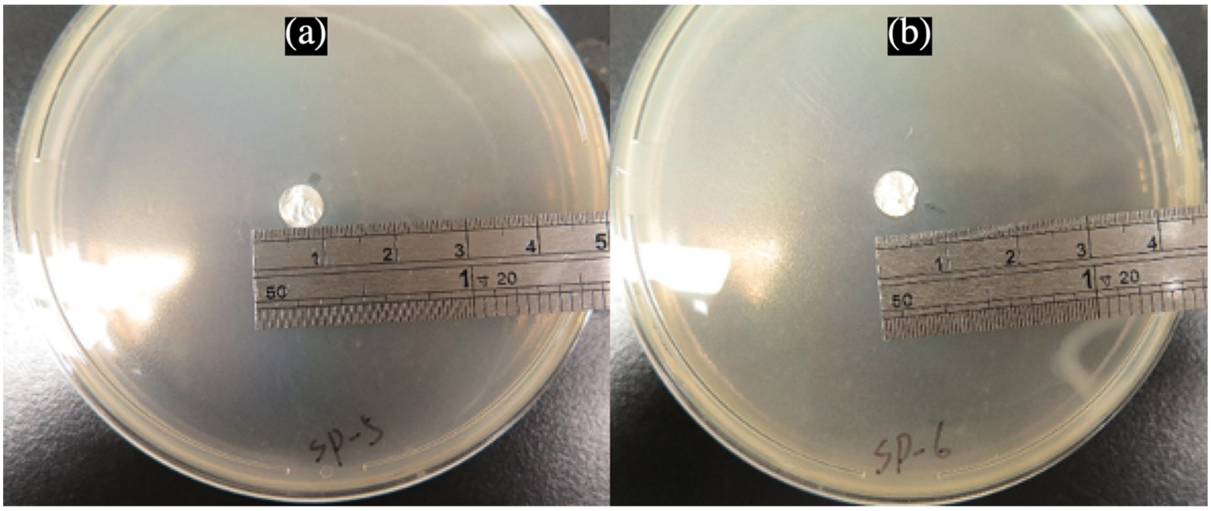

Figure S6. (**a**) 10% PAN-CuNP Nanofiber Disk and (**b**) 15% PAN-CuNP Nanofiber Disk ZOI Tests on *S. pneumoniae*

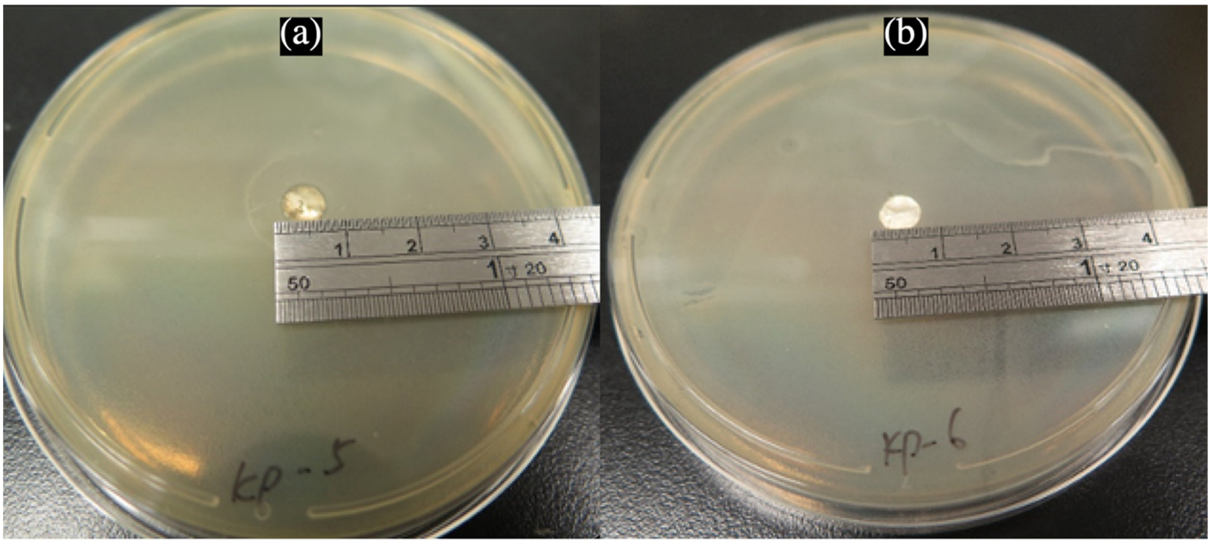

Figure S7. (**a**) 10% PAN-CuNP Nanofiber Disk and (**b**) 15% PAN-CuNP Nanofiber Disk ZOI Tests on *K. pneumoniae*

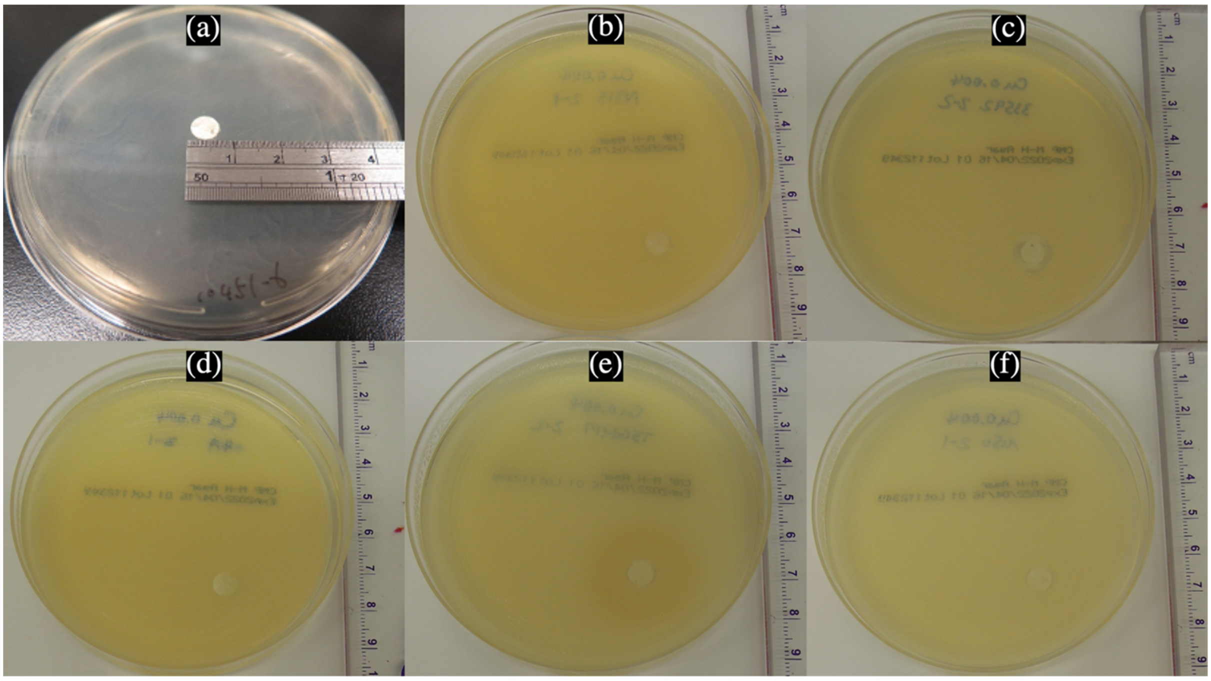

Figure S8. 15% PAN-CuNP Nanofiber Disk ZOI Tests on (**a**) MRSA (*10451*), (**b**) MRSA SCC*mec*II (*N315*), (**c**) MRSA SCC*mec*III (*33592*), (**d**) MRSA SCC*mec*IV (*IVa*), (**e**) MRSA SCC*mec*V_T (*TSGH17*), and (**f**) VISA ($\mu 50$)

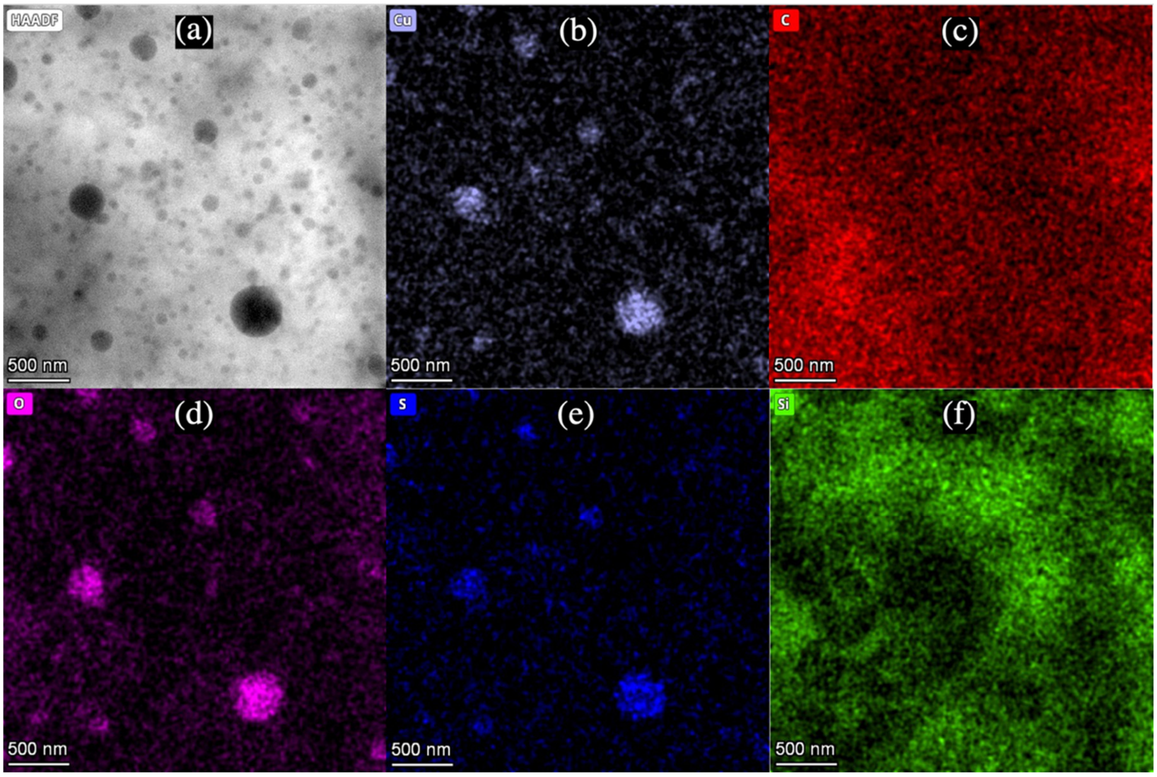

Figure S9. (**a**) HAADF-TEM Image of PAN/DMF/CuNP Colloidal Solution. (**b**) Copper, (**c**) Carbon, (**d**) Oxygen, (**e**) Sulfur, and (**f**) Silicon Elemental Distribution in TEM Image of PAN/DMF/CuNP Colloidal Solution

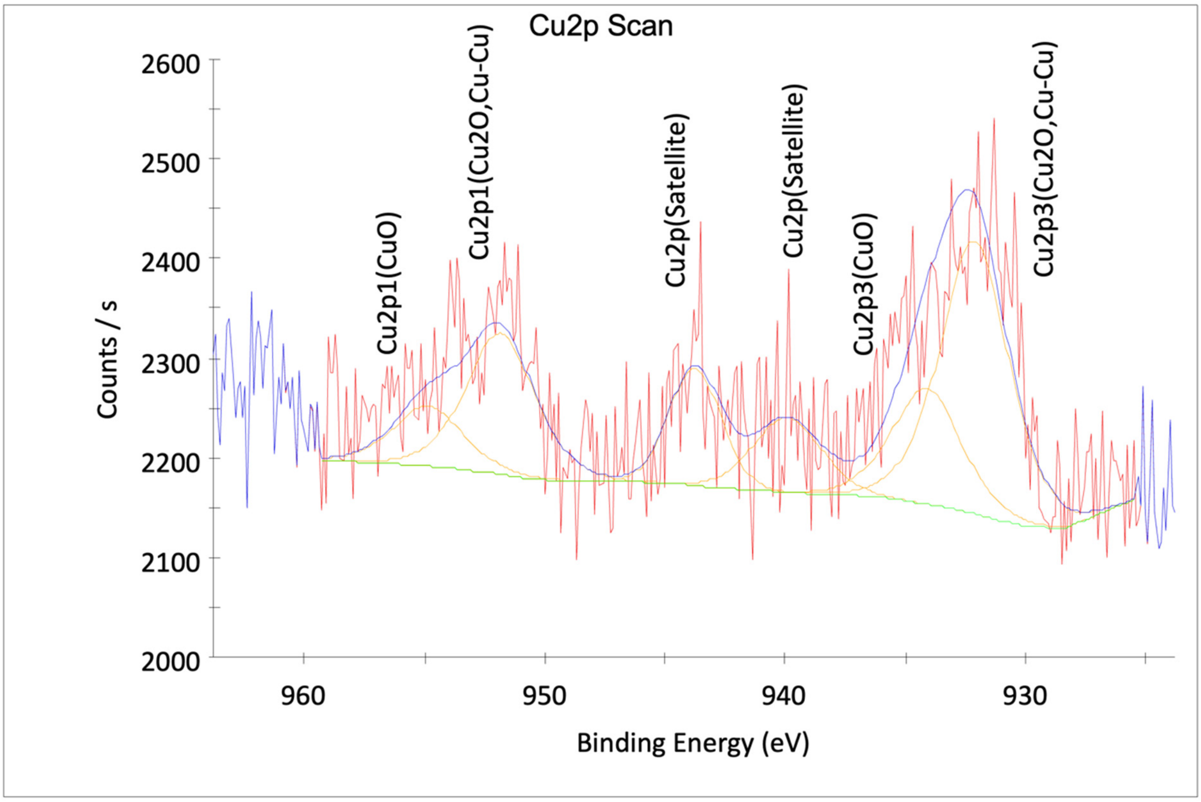

Figure S10. Cu2p XPS Core-Level Spectra of 15% PAN-CuNP Nanofiber Sample

Table S1. Cu2p XPS Atomic Weight Quantification of 15% PAN-CuNP Nanofiber Sample

XPS Detection Name	Atomic Weight Concentration (%)
**Cu2p3 (Cu_2O, Cu-Cu)**	**70.1**
**Cu2p3 (CuO)**	**29.9**

Figure S11. Homogenized 15% PAN/DMF/CuNP Solution with Dark Green Hue

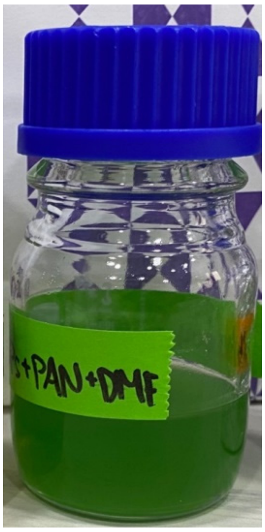

Supplement: Supplementary file 1 [file nanomaterials-12-02139-s001.zip › nanomaterials-1749105-supplementary.pdf]
